# Isolation and Characterisation of Major and Minor Collagens from Hyaline Cartilage of Hoki (*Macruronus novaezelandiae*)

**DOI:** 10.3390/md17040223

**Published:** 2019-04-12

**Authors:** Mathew H. Cumming, Bronwyn Hall, Kathleen Hofman

**Affiliations:** Seafood Research Unit, The New Zealand Institute for Plant and Food Research Limited, P.O. Box 5114, Port Nelson 7043, New Zealand; Bronwyn.hall@plantandfood.co.nz (B.H.); Jacqui.Day@plantandfood.co.nz (K.H.)

**Keywords:** type II collagen, type IX collagen, type XI collagen, cartilage, marine, fish, arthritis

## Abstract

The composition and properties of collagen in teleost (bony fish) cartilage have never been studied. In this study, we aimed to identify and characterise all collagen species in the nasal cartilage of hoki (*Macruronus novaezelandiae*). Four native collagen species were extracted using two techniques, and isolated with differential salt precipitation. We were able to assign the identity of three of these collagen species on the basis of solubility, SDS-PAGE and amino acid analyses. We found that hoki cartilage contains the major collagen, type II, and the minor collagens, type IX and type XI, which are homologous to those found in mammal and chicken cartilage. Using these extraction protocols, we also isolated a full-length type IX collagen from cartilage for the first time. In addition, we detected a 90 kDa, highly glycosylated collagen that has not been identified in any other species. For each isolate, structural and biochemical characterisations were performed using circular dichroism and Fourier transform infrared spectroscopy analyses, and the thermal denaturation properties were determined. Our results showed that the properties of hoki cartilage-derived collagens are similar to those of collagens in mammalian cartilage, indicating that teleost cartilage could provide biological ingredients for the development of biomaterials to treat cartilage-related illnesses.

## 1. Introduction

Collagens and proteoglycans are the main proteins in the extra cellular matrix of cartilage and, together with water, they give cartilage its strength and elastic properties [1]. In human cartilage, the majority of the collagen framework is made up of type II collagen, accompanied by lesser amounts of minor collagens (1–6% of total collagen), such as type IX and XI collagens [2]. Type II collagen is a fibril-forming collagen found exclusively in cartilage, vitreous humour, and the notochord. It is a homotrimeric molecule consisting of three identical alpha chains ([α1(II)]_3_), each approximately 120 kDa. Type XI collagen is a heterotrimeric fibril-forming collagen with three distinct alpha chains: α1(XI), α2(XI) and α3(XI)] [3]. The α3(XI) chain has the same primary sequence as α1(II) but differs in its degree of glycosylation. Little is known about type XI collagen. It is believed to play a key role in initiating fibrillogenesis and its presence in the fibril has been shown to restrict fibril width [4]. The longer α1(XI) chain contains an N-terminal non-collagenous domain with sequence homology to the TSPN domain of thrombospondin [5,6], which binds glycosaminoglycans [7] and plays a role in organisation of the extracellular matrix.

Type IX collagen is a member of the FACIT (fibril-associated collagens with interrupted triple helices) family. It comprises three different alpha chains (α1(IX), α2(IX), α3(IX)), each made up of three triple helical collagenous (COL) domains (COL1–3) and four non-helical non-collagenous (NC) domains (NC1–4). Each of the alpha chains of type IX collagen is crosslinked between lysine residues and with the C-terminal region of type II collagen [2,8]. The type IX triple helix is organized on the outside of the collagen fibril, where the NC3 domain acts as a hinge to project the COL3 domain and a laminin-neurexin-sex hormone binding globulin (LNS) domain [9] away from the fibril surface [10]. This orientation facilitates binding to glycosaminoglycans, fibronectin and cartilage oligomeric matrix protein (COMP) [11,12,13].

There is a growing need for the development of cartilage scaffolds for the treatment for cartilage trauma, osteoarthritis and other cartilage-related illnesses. This has led to an increasing demand for cartilage replacement scaffolds and a better understanding of the types and properties of collagens within cartilage [14,15]. The purpose of this study was to identify and characterise the major and minor collagens of teleost cartilage. Knowledge about the collagens in teleost cartilage is very limited, as most previous studies have focused on collagens in cartilage sourced from mammals. In this study, we used the nasal cartilage from a fish, hoki (*Macruronus novaezelandiae*). This species makes up the largest tonnage in the New Zealand fishery and represents a significant by-product resource. We isolated the putative major (type II) and minor collagens (type IX and XI) from pepsin- and alkaline-solubilised hoki cartilage using the differential salt precipitation method. The collagen species were identified by amino acid and electrophoretic analyses. Their secondary structure and thermal denaturation properties were determined by circular dichroism (CD) analyses, and their chemical properties were investigated by Fourier transform infrared (FTIR) spectroscopy. These extraction methods and the characterization of major and minor collagens will lead to a better understanding of the collagen composition of teleost cartilage, which may be a suitable bioresource for the development of products to treat cartilage-related diseases.

## 2. Results

### 2.1. Extraction and Isolation of Cartilage Collagens

**Figure 1:**
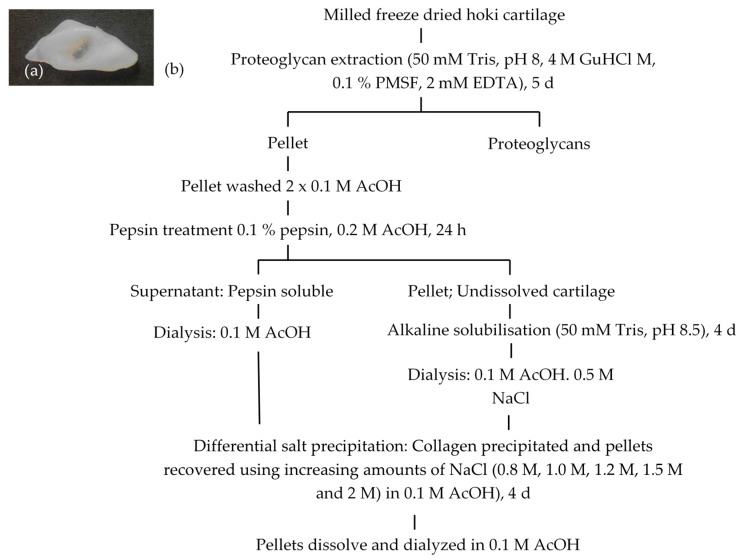
(**a**) Nasal cartilage from hoki; (**b**) flow diagram for extraction of collagens from hoki cartilage.

Figure 1 shows the flow chart for the extraction and isolation of collagen species from hoki cartilage. Proteoglycans were removed using guanidinium hydrochloride (GuHCl), which was necessary for effective protein isolation from cartilage. Collagen species were then extracted using limited pepsin digestion. No protein was detected by sodium dodecyl sulphate polyacrylamide gel electrophoresis (SDS-PAGE) or Biuret assay after 0.5 M acetic acid treatment (data not shown). After the pepsin treatment, the soluble fraction was recovered by centrifugation (pepsin-soluble fraction). Multiple pepsin treatments of the insoluble fraction did not fully dissolve the cartilage. Interestingly, subsequent treatment of the insoluble fraction with 50 mM Tris (pH 8.5) quickly dissolved the pepsin-treated cartilage; hereafter, this fraction is referred to as the alkaline-soluble fraction.

Different native collagen species were isolated from the pepsin- and alkaline-soluble fractions using the salt precipitation method [16]. To ensure isolation of single species, the salt precipitation process was repeated.

### 2.2. Isolation of Cartilage Collagens Using Differential Salt Precipitation and SDS-PAGE Analysis

**Figure 2:**
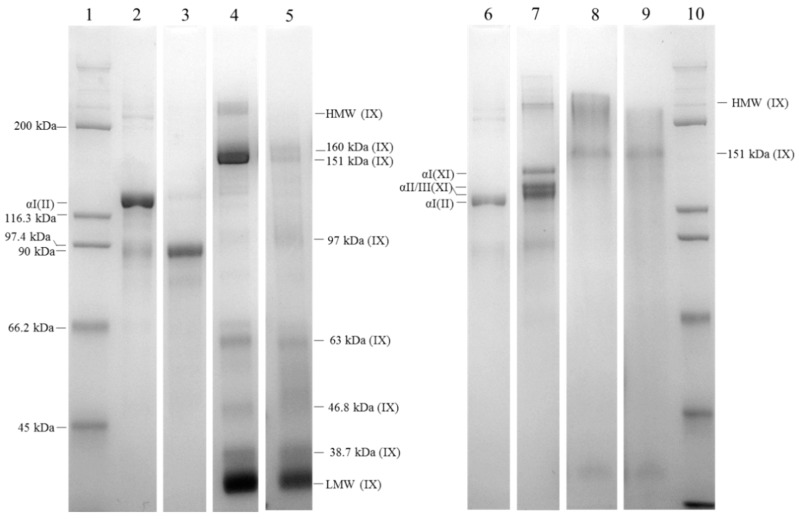
SDS-PAGE analysis of differentially salt (NaCl)-precipitated collagens from hoki cartilage. Lanes 2–5, from pepsin-soluble fraction. Lanes 6–9, from alkaline-soluble fraction. Lanes 1 and 10, Broad range marker; lanes 2 and 6, proteins precipitated by 0.8 M NaCl; lane 3, proteins precipitated by 1.0 M NaCl; lane 7, proteins precipitated by 1.2 M NaCl; lanes 4 and 8, proteins precipitated by 2 M; lanes 5 and 9, same proteins as in lanes 4 and 8 after incubation with a reducing agent.

The salt-precipitated fractions from the pepsin- and alkaline-soluble extracts were analysed by SDS-PAGE (Figure 2). In this study, we compared the molecular weight profile of the isolated proteins and their response to a reducing agent with information reported in the literature, to determine their identity.

**Figure 3:**
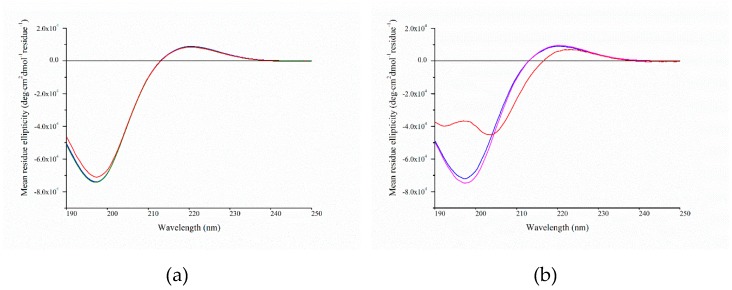
Circular dichroism wavelength spectra of (**a**) pepsin-soluble hoki samples: Type II collagen (**━━**), 90 kDa protein (**━━**), Type IX (**━━**); (**b**) alkaline-soluble samples: Type II collagen (**━━**), Type XI (**━━**), Type IX (**━━**).

For both soluble fractions, precipitation with 0.8 M NaCl resulted in one major band at ~128 kDa, consistent with type II collagen from terrestrial sources. This type of collagen was homomeric, comprising three identical α-chains (Figure 3, lanes 2 and 6) [17,18]. Increasing the salt concentration in the pepsin-solubilized fraction to 1.0 M precipitated a single 90 kDa protein (lane 3). This protein precipitated at a salt concentration close to that used to precipitate type II collagen, so it was often difficult to isolate one from the other. Based on the molecular weight and the salt concentration at which it precipitated, it was difficult to identify this protein from the literature. To determine whether the 90 kDa protein was a result of pepsin cleavage of type II collagen, we digested type II collagen with pepsin for an extended period. The digestion products did not include a 90 kDa protein, indicating that this protein did not result from pepsin cleavage of type II collagen (Supplementary data, Supplementary Figure S1).

We expected that 1.2 M NaCl would precipitate type XI collagen [16], but we only achieved recovery of protein from the alkaline-soluble fraction. The precipitated proteins consisted of three major bands at ~144, ~136 and ~128 kDa with similar densities (Figure 3, lane 7). The banding pattern and molecular weights were consistent with those reported for the three alpha chains of type XI collagen isolated from human [18], bovine [19], chicken [20,21] and lamprey [22].

According to Deyl (2003), 2.0–3 M NaCl should precipitate type IX collagen. Separation of the pepsin-treated sample by non-reducing SDS-PAGE yielded a light band at 200 kDa, a dense band at 151 kDa, and a dense band at 33.4 kDa, as well as light bands at 63, 46.8 and 38.7 kDa (Figure 3, lane 4). These results are consistent with those of other studies on type IX collagen [23]. In previous reports, pepsin-solubilised type IX collagen separated into two bands in SDS-PAGE analyses under unreduced conditions: a high molecular weight band (HMW) (~225 kDa) consisting of the three alpha chains crosslinked by disulphide bonds, and a low molecular weight (LMW) band corresponding to the COL1 domain (~ 30 kDa) [20,23,24,25,26]. Adding a reducing agent dissolved the HMW band to form multiple bands caused by pepsin proteolysis (Figure 3, lane 5), a pattern that has also been observed for mammalian and chicken type IX collagen [23,24]. For hoki, light bands consistent with these fragments were also present without adding a reducing agent, suggesting the absence of disulphide bonds between some of the fragments. After reduction of the hoki sample, we observed a marked decrease in the density of the HMW bands at 200 and 150 kDa and new bands at 160 and 97 kDa.

Similar results were obtained for the alkaline-soluble fraction, with bands at 200, 151 and 38.7 kDa. However, compared with the equivalent sample from the pepsin-soluble fraction, there was a lower proportion of the 200 kDa protein and the band was more diffuse. The absence of the lower molecular weight fragments (at 63, 46.8 and 38.7 kDa) was consistent with extraction without pepsin. When this sample was reduced, there was less of the 200 kDa protein band and no LMW protein bands. These results and those of the circular dichroism spectra suggest that this isolate represents the full-length type IX collagen.

### 2.3. Amino Acid Analysis

Table 1 shows the amino acid composition of the collagen species isolated from cartilage. All samples had different amino acid compositions. The values for all the samples were within the ranges expected for members of the collagen family, with large amounts of glycine and imino acids (proline and hydroxyproline) (277–299.3 and 183.21–240.12/1000 residues, respectively) [27]. Compared with the other samples, the 90 kDa protein showed similar proportions of glycine and imino residues, suggesting that it is a member of the collagen protein family.

Comparison of the amino acid composition of hoki cartilage collagens and reported data from other species are in Supplementary Tables S1–S3. The overall composition of type II collagen from hoki cartilage was similar to that of type II collagens in the cartilage and notochord of other species including chicken (sternum) [28], bovine [20,22], lamprey [22] and skate [29]. The composition of hoki pepsin- and alkaline-soluble type IX collagen was similar to that isolated from chicken and bovine cartilage, but had approximately 10% less hydroxyproline [20,30]. The amino acid composition of type XI collagen from hoki cartilage was more similar to type XI collagen in human than to type XI collagen in lamprey [22,31].

Comparing the pepsin- and alkaline-soluble type IX collagen samples, we observed that the alkaline-soluble form had lower proportions of collagen-related residues, such as glycine, alanine, and proline, and higher proportions of glutamate, valine, isoleucine, leucine and hydroxyproline. These results complemented the circular dichroism and FTIR data, which suggested that alkaline-soluble type IX collagen from hoki contains non-collagenous protein domains.

In theory, the cysteine residues should have been destroyed during sample preparation [32]. However, we detected some cysteine residues in type IX collagen extracted from both the pepsin- and alkaline-soluble fractions, consistent with the detection of disulphide bonds by SDS-PAGE analysis.

### 2.4. Circular Dichroism Spectroscopy

We used CD spectroscopy to assess the secondary structure of cartilage proteins. The collagen molecule gives distinct CD spectra with a large negative peak at 197 nm and a rise at 220 nm attributed to the unique left-handed polyproline-type II helix [33,34]. Wavelength scans for each sample are shown in Figure 3. From the pepsin-soluble fraction, isolated type II and type IX collagens and the unidentified 90 kDa protein had a negative peak at 197 nm and a positive peak at 220 nm, typical of native triple-helix collagen. The type II and XI collagens isolated from the alkaline-soluble fraction had similar spectra, confirming their identities as native collagen. Unexpectedly, the spectra for type IX collagen isolated from the alkaline-soluble fraction had a positive peak at 223 nm and two negative peaks at 192.4 and 203 nm, suggesting that, unlike the other isolated collagens, this sample contained other secondary structural features.

Accurately estimating the secondary structure of proteins containing collagen domains is challenging [35]. Using the Dichroweb server to estimate secondary structure, we compared the CD spectra of pepsin-soluble type II collagen and alkaline-soluble type IX collagen [36]. Compared with type II collagen, type IX collagen had a larger proportion of beta sheets (+14%) and a lower proportion polyproline type II helices (−10%). The high proportion of beta sheets is consistent with reports that the NC4 domain of the α1 of type IX collagen has sequence and structural homology to the beta sheet-rich laminin-neurexin-sex hormone binding globulin (LNS) domain, which associates with glycosaminoglycans [9,37].

The differences in secondary structure between type IX collagen isolated from the pepsin-soluble and alkaline-soluble fractions suggested that there are two isoforms, one with and one without an NC4 domain, as shown in chicken [38,39]. Alternatively, the NC4 domain may have been cleaved during pepsin treatment.

### 2.5. Thermal Denaturation Analyses Using Circular Dichroism Spectroscopy

**Figure 4:**
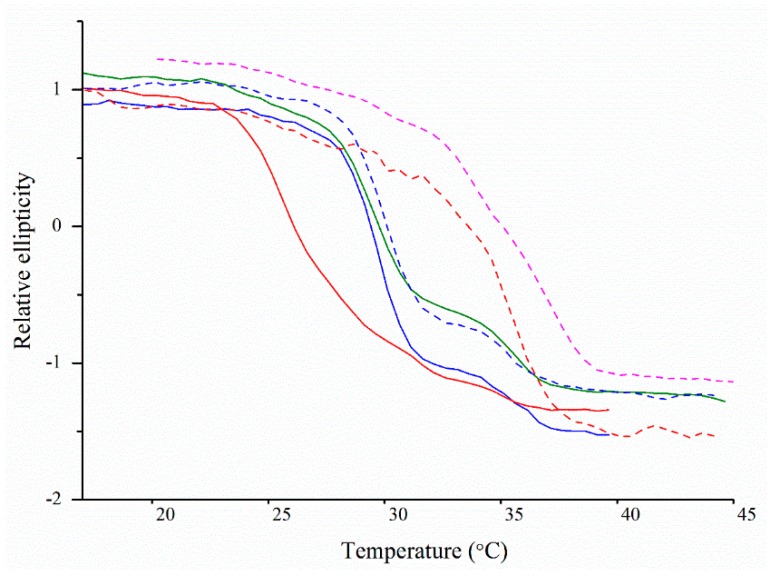
Thermal melting curves of collagens extracted from hoki cartilage. Pepsin-soluble type II collagen (**━━**), type IX (**━━**), 90 kDa (**━━**). Alkaline-soluble type II collagen (**┅**), type XI (**┅**), type IX (**┅**).

The CD absorbance spectra were monitored for the isolated collagen samples with a heating rate of 1 °C/min to monitor unfolding (Figure 4) and to determine the thermal melt temperatures (*T*_m_). The melting curves for both type II collagen preparations were identical, with unfolding occurring in two events before denaturation. In the first event, ~70% of protein unfolded with a *T*_m_ of ~30.1 °C, and in the second event, protein unfolding occurred between 33 and 36 °C, but the difference was too small to determine the *T*_m_ accurately. The 90 kDa protein showed a similar unfolding intermediate at the same temperature range (33–37 °C) and a slightly lower *T*_m_ of 29.4 °C. Type XI collagen showed a slower melting transition, which gave the highest *T*_m_ in this study (35.9 °C), 5.9 °C higher than that of type II collagen, the other fibrillar collagen.

The thermal melt curve for type IX collagen isolated from the alkaline-soluble fraction showed a slow melt, from approximately 25 to 34 °C, then a quick transition with a *T*_m_ of 35.36 °C. Measuring the absorbance at 203 nm gave a similar kinetic profile and a *T*_m_ of 35.56 °C (data not shown). In contrast, the *T*_m_ of type IX collagen from the pepsin-soluble fraction was 9.1 °C lower (26.3 °C). The melt curve was also different, with a gradual transition and signs of multiple transitions.

Hydroxyproline residues are known to stabilize collagen and increase the thermodenaturation temperature [40]. The hydroxyproline values of the various samples are shown in Table 1. We did not observe a clear relationship among the samples that displayed multiple-stage melting curves (observed for type II and 90 kDa protein samples); however, this relationship was observed for the type IX and type XI collagen samples (Figure 4). In this study, pepsin-soluble type IX and alkaline-soluble type XI had the lowest and highest measured hydroxyproline values (85.9 residues/1000 residues and 105.33 residues/1000 residues, respectively), which corresponded with the lowest and highest *T*_m_ values (26.3 °C and 35.9 °C) (Figure 4 and Table 1).

Comparisons with previously reported thermodenaturation temperatures for type II collagen in other species showed that the first and largest unfolding for hoki type II collagen was lower than those of type II collagens from the notochord and cartilage of sturgeon (36.3 and 35.7 °C, respectively) [41,42] and from bovine cartilage and chicken sterna articular cartilage (42.8 and 43.8 °C, respectively) [20,37]. Since the thermal denaturation temperature of collagens is known to be related to the habitat of the organism [28,43], the lower *T*_m_ values of hoki collagens are consistent with the cold-water habitat of this fish species.

Morris et al. (1990) showed that bovine type XI collagen melted in two transitions, at 38.5 and 41.5 °C, and the *T*_m_ of type XI collagen was 2.7 to 4.3 °C lower than that of type II collagen [43]. In contrast, we found that the *T*_m_ of type XI collagen was higher than that of type II collagen from hoki. Bruckner et al. (1983) investigated the thermal properties of pepsin-soluble type IX collagen from chicken, and observed multi-step unfolding transitions (31, 37 and 44 °C) at higher temperatures than those observed for pepsin-soluble type IX collagen from hoki [44].

### 2.6. Fourier Transform Infrared Spectroscopy

**Figure 5:**
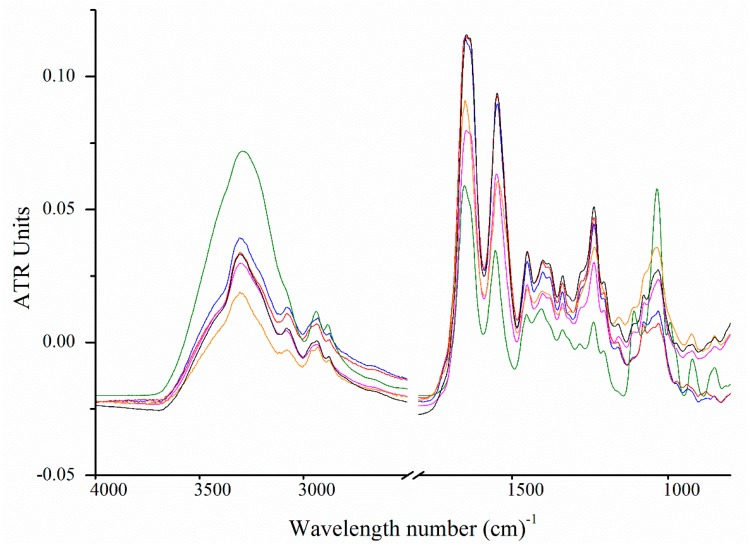
Fourier transform infrared spectra of collagens isolated from hoki cartilage. Pepsin-soluble type II collagen (**━━**), type IX (**━━**), 90 kDa (**━━**). Alkaline-soluble type II collagen (**━━**), type XI (**━━**), type IX (**━━**).

The FTIR absorbance can be used to detect the presence of chemical bonds and describe the tertiary structure of collagen. Figure 5 compares the FTIR spectra of the isolated hoki cartilage collagen samples. Peaks corresponding to amide A (~3304 cm^−1^), amide B (~3075 cm^−1^), amide I (~1650 cm^−1^), amide II (1550 cm^−1^) and amide III (~1237 cm^−1^) were present in the spectra of all samples, consistent with patterns observed in spectra for collagen from other sources, including type II collagen from chicken sternum [45,46].

**Figure 6:**
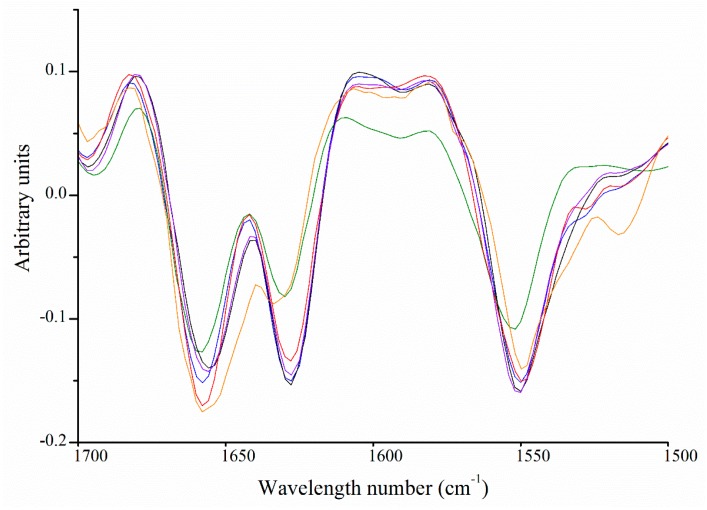
Second derivative Fourier transform infrared spectra of collagens isolated from hoki cartilage between 1500 and 12,700 cm^−1^. Pepsin-soluble type II collagen (**━━**), type IX (**━━**), 90 kDa (**━━**). Alkaline-soluble type II collagen (**━━**), type XI (**━━**), type IX (**━━**).

The amide I absorption peak (1690–1580 cm^−1^) arises from the C=O stretch of amides (70%–85%) and C–N stretching (10%–20%). The exact frequency depends on the secondary structure of the protein backbone and; thus, is the most informative for secondary structure analysis [47,48]. Spectra from all samples showed a double or flattened peak, indicating two or more overlapping peaks. To reveal these peaks, we applied the Savitzky–Golay second derivative algorithm (Figure 6). All samples had strong absorbance at ~1660 cm^−1^ and ~1627 cm^−1^, which were associated with native triple-helical collagen and water-backbone associations, respectively, consistent with the CD data (Figure 3) [46,47,49,50]. Type IX collagen from the alkaline-soluble fraction showed the greatest difference in amide I band with a broader 1660 cm^−1^ peak and a red-shift of the ~1627 cm^−1^ peak to 1631 cm^−1^. This indicated greater diversity in the conformation of the protein backbone as a result of different secondary structures [51,52]. These results supported the different secondary structures detected in the CD analysis (Figure 3).

#### Carbohydrate Spectral Region (from 1200 to 850 cm^−1^)

**Figure 7:**
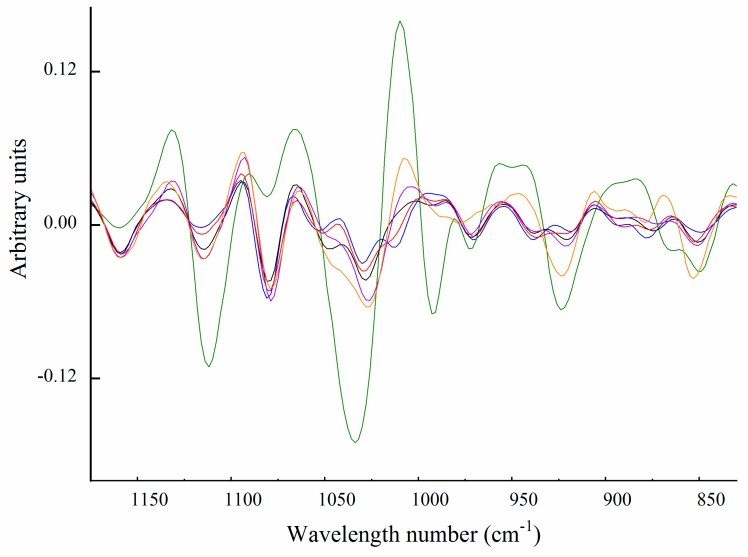
Second derivative Fourier transform infrared spectra of collagens isolated from hoki cartilage between 825 and 1200 cm^−1^. Pepsin-soluble type II collagen (**━━**), type IX (**━━**), 90 kDa (**━━**). Alkaline-soluble type II collagen (**━━**), type XI (**━━**), type IX (**━━**).

The FTIR spectral data between 1200 and 850 cm^−1^ were processed using second derivatization (Figure 7). All samples showed absorbance at ~1076 and 1036 cm^−1^ (Figure 7), which could be attributed to the C–O stretching of galactose or glucose-galactose groups [53,54]. Carbohydrate-associated absorption bands were most apparent in type IX collagen isolated from the alkaline-soluble fraction and the unidentified 90 kDa protein. Type IX collagen had additional absorbance peaks at 920 cm^−1^ and 850 cm^−1^, which have previously been attributed to the S–O stretching of glycosaminoglycans [55], consistent with previous reports that chondroitin sulphate is covalently attached to type IX collagen [39,56,57]. The 90 kDa protein isolated from the pepsin-soluble fraction showed very strong absorbance in this region. Large absorption bands at 1111, 1034, 991, 920 and 850 cm^−1^ suggested a very high degree of glycosylation with a sulphated glycosaminoglycan [45]. We could not confidently assign the 1111 and 991 cm^−1^ absorption bands, but they are likely to be related to glycosaminoglycans [55]. The absorption band at 1111 cm^−1^ may arise from the anti-symmetrical C–O–S stretching at 1124 cm^−1^ reported for chondroitin sulphate [58].

## 3. Discussion

Collagen fibrils play an integral role in cartilage, contributing to the structural and signalling messages that maintain healthy cartilage [59,60]. The Identification and characterisation of the major and minor collagens that make up fibrils in terrestrial animals are well established [61]. However, there have been no efforts to understand the composition or characteristics of collagens within the cartilage of teleost (bony fish), and limited research on cartilage-derived collagens from other marine species [22,41,42]. There has been a surge in the development of biomaterials for cartilage replacement to treat osteoarthritis and other cartilage-related diseases. For this to be successful, it essential to have a reliable source of well-characterised major and minor collagens that mimic human collagens.

In the present study, we found that we could extract and purify type II collagen and the minor type IX and XI collagens from hoki cartilage. Hoki cartilage comprises type II collagen and the minor type IX and XI collagens with homologous α chain assembly, amino acid composition and structure to those of collagens found in cartilage of mammals and chickens [61,62]. Moreover, we detected a 90 kDa protein that could not be assigned based on the current literature. Our results showed that this was highly glycosylated and had structural and amino acid composition consistent with a collagen molecule. Based on its similarities to type II collagen, we believe that the 90 kDa protein is a fibrillar, glycosylated, shorter isoform of type II collagen that has a role in associating with proteoglycans to maintain cartilage integrity. To understand its identity and role, further research to determine the gene and protein sequence, and post-translation modifications should be performed.

To isolate all collagen types, it was necessary to use two extraction methods: limited pepsin digestion, previously reported to cleave the telopeptides of type II collagen and sensitive regions of type IX collagen; and alkaline extraction, to solubilize type XI and full-length type IX collagens. The collagen samples were then purified using differential salt precipitation. Using these methods, we were able to isolate native collagen with high purity. We suspect that alkaline treatment was necessary to alter the charge of the remnant glycosaminoglycans which bound the collagen molecules as an insoluble matrix. This increase in pH resulted in repulsive forces that dissolved the matrix and resulted in the solubility of the remaining collagen. This method allowed us to extract a full-length type IX collagen from cartilage for the first time. Analyses of amino acid composition and structure using circular dichroism suggested a high proportion of beta-sheets, consistent with an LNS domain, which associates with glycosaminoglycan and is integral to matrix integrity [9,37]. If this is the case, then type IX collagens are likely to play similar roles in both fish and mammalian cartilage.

Analysis of the thermal-denaturation properties showed that three collagens (pepsin- and alkaline-soluble type II, 90 kDa collagen, and alkaline-soluble type IX) unfolded in multiple-stages. This can be explained by either the protein sample containing multiple species, as the case for pepsin-soluble type IX collagen, or multiple unfolding events. This has been reported previously for pepsin-soluble type I collagen isolated from various marine species [63,64], human type V collagen, and bovine type XI collagen [43]. Miles and Bailey (1999, 2001) proposed that unfolding does not occur simultaneously; instead, regions with less hydroxyproline residues are more thermally labile and unfold first [65,66]. It was also interesting to observe that loss of the an LNS domain in type IX caused a 9.1 °C drop in *T*_m_, and a slower unfolding rate. This could be explained by this domain either having a higher *T*_m_ or that it stabilizes the collagen domain.

Type II collagens have been isolated from the cartilage and notochord of other marine vertebrates, including sturgeon and lamprey [22,41,42]. Some of these studies have also identified type XI collagens. Members of the elasmobranchii subclass (sharks and skates) are renowned for their cartilaginous skeletons. However, evidence from many species is that the skeleton contains mostly type I collagen [67,68,69]. The only exception is the pectoral fin of skate, which also contains type II collagen [29]. Based on the literature, the collagen makeup of teleost cartilage is likely to be closer to those of sturgeon, lamprey, and terrestrial species than to those of sharks and skates. It has been well established that type I collagen from marine species has lower thermal properties, and hydroxyproline values than terrestrial species. Generally speaking, we observed this for cartilage-derived collagens. This difference was most pronounced for pepsin soluble type IX. When compared with chicken type IX, hoki type IX showed 10% less hydroxyproline and ~30% lower *T*_m_ [44].

Type II collagen scaffolds or hydrogels are showing promise in biomedical applications for cartilage repair and replacement [56,70,71,72,73]. Future studies investigating if human type II antibodies recognize hoki type II would be advantageous. We see potential with the integration of type IX or XI collagen into these biomaterials. Despite the known importance of minor collagens in cartilage development, this has never been done [7,11,61,74]. It is possible that this is due to the lack of commercial supply.

Overall, hoki cartilage comprises type II, IX and XI collagens with similar biochemical characteristics to those of their counterparts in mammals. Assessment of the biocompatibility and immune response of these collagens with human cells is yet to be conducted, but hoki cartilage holds great promise as a source of suitable ingredients for valuable biomaterials.

## 4. Materials and Methods

### 4.1. Chemicals

Acetic acid, Tris base, sodium hydroxide, and sodium chloride were purchased from Thermo Fisher Scientific Ltd. (Auckland, New Zealand). Copper sulfate, sodium dodecyl sulfate (SDS), pepsin (porcine gastric mucosa >250 U/mg, Sigma P 7000), GuHCl, ethylenediaminetetraacetic acid (EDTA), and phenylmethylsulfonyl fluoride (PMSF) were purchased from Sigma-Aldrich (St. Louis, MO, USA).

### 4.2. Materials

Hyaline nasal cartilage was dissected from hoki (*Macruronus novaezelandiae*) obtained from a seafood processing company (Sealord, Nelson, New Zealand). Cartilage was washed in 0.5 M acetic acid for 24 h at 8 °C, to remove extraneous ligaments, and then freeze-dried and milled. On average, cartilage from whole hoki noses measures approximately 3 × 1.5 × 0.5 cm.

### 4.3. Collagen Extraction from Cartilage

Unless stated, materials were prepared in a temperature-controlled room set at 8 °C. Glycosaminoglycans were removed from the tissue by two successive extractions in 50 mM Tris-HCl (pH 8), 4 M GuHCl, 0.1% PMSF, and 2 mM EDTA for 5 days. Tissue was recovered by centrifugation (3260× *g*, 15 min, 4 °C) and washed twice with 0.1 M acetic acid. Cross-linked collagens were solubilized by digestion with 0.1% pepsin in 0.2 M acetic acid for 24 h at 8 °C. The soluble fraction was recovered by centrifugation at 3260× *g*, and the pH was briefly increased to 8.5 for 20 min. The pepsin-soluble fraction was dialyzed against 0.1 M acetic acid. The remaining cartilage was solubilized by two successive extractions in 50 mM Tris-HCl (pH 8.5) for 4 days. The dissolved cartilage (alkaline-soluble fraction) was dialyzed against 0.1 M acetic acid containing 0.5 M NaCl.

### 4.4. Collagen Isolation Using Salt Precipitation

Different types of collagen were isolated from the pepsin- and alkaline-soluble fractions using a modified salt precipitation method, with concentrations based on Deyl (2003) [16]. Proteins were precipitated by mixing each extract with increasing concentrations of NaCl (0.8 M, 1.0 M, 1.2 M, 1.5 M, and 2 M) and incubating the mixture at 8 °C for 2 h. Between each NaCl step, the precipitated protein was collected by centrifugation (76,400× *g*, 50 min, 4 °C). The supernatant was used for the next NaCl step. The pellet was dissolved in 0.1 M acetic acid, and subsequently dialyzed against 0.1 M acetic acid to remove NaCl. To improve purity, isolates were re-precipitated using the same salt concentration.

### 4.5. Sodium Dodecyl Sulfate Polyacrylamide Gel Electrophoresis Analyses

For SDS-PAGE analyses, samples were mixed with 2 × loading buffer (125 mM Tris-HCl, pH 6.8, 20% glycerol, 4% SDS, 0.1% bromophenol blue) according to Laemmli (1970) [75]. Reduced samples were incubated in 50 mM DTT at 40 °C for 30 min prior to mixing with 2 × loading buffer containing 5% mercaptoethanol. Samples were loaded onto a 7.5% acrylamide gel (Criterion, BioRad, Hercules, CA, USA) and visualized after staining with BioSafe™ (BioRad, Hercules, CA, USA). The molecular weights of protein bands were determined by comparison with a broad range marker (BioRad, Hercules, CA, USA).

### 4.6. Amino Acid Derivatization and Analysis

Amino acid composition was determined using an ultra-high-performance liquid chromatograph (UHPLC; Dionex Ultimate 3000 series, Dionex, Sunnyvale, CA, USA) equipped with a Kinetex^®^ 2.6 µm EVO C18 (150 × 3.0 mm) column (Phenomenex, Torrance, CA, USA). Samples were hydrolysed for 24 h in 6 N HCl at 110 °C and derivatized with o-phthalaldehyde (OPA) and 9-fluorenylmethyl chloroformate (FMOC) [76]. Absorbances at 338 and 266 nm were monitored and compared against standard curves of derivatized standard amino acid mixes (Agilent, Palo Alto, CA, USA) and derivatized hydroxyproline and hydroxylysine (Sigma, St. Louis, MO, USA). Data were processed using Chromeleon 7.2 SR4 software (Dionex, Sunnyvale, CA, USA).

### 4.7. Biuret Assay

The protein concentration was measured using the Biuret assay [77] with a collagen standard curve prepared from purified type I collagen from hoki skin [46]. Type I hoki collagen was chosen as a standard because it could be prepared with high purity in large quantities.

### 4.8. Circular Dichroism Analyses

The CD spectra were collected using a Jasco 1500 CD spectrometer (Jasco Inc., Easton, MD, USA) from samples in 1-mm quartz cuvettes (Starna, Atascadero, CA, USA). Protein solutions were prepared at a concentration of 0.2 mg/mL in 1 mM acetic acid. Wavelength scans were repeated five times at 8 °C and the data were accumulated for each sample. Scans were measured between 190 and 250 nm with a bandwidth of 1 nm at a scanning speed of 20 nm/min. Thermal denaturation studies were performed by monitoring the ellipticity at 220 or 223 nm (for type IX collagen isolated from the alkaline fraction) between 15 and 45 °C at a heating rate of 1 °C/min. Measurements were made every 0.5 °C with a 5 s equilibration time. All data were subtracted from baseline measurement of 1 mM acetic acid. Jasco Spectral manager software (version 2.14, Jasco Inc., Easton, MD, USA) was used for data processing. Spectra were smoothed using a Savitsky–Golay filter.

Secondary structure was estimated using the program SELCON3 with protein reference set to 5 using the Dichroweb Server (http://dichroweb.cryst.bbk.ac.uk/html/home.shtml) [37,78]. To convert the ellipticity to mean residue ellipticity, the mean residue weights of all samples were calculated as 95.35. This was based on the alpha 1 (II) as reported on the Uniprot website (entry P02458; https://www.uniprot.org/).

### 4.9. Fourier Transform Infrared Spectroscopy

The FTIR experiments were carried out using a Bruker Alpha FTIR spectrometer (Bruker Optik GmbH, Ettlingen, Germany). Freeze-dried samples were clamped onto the crystal using an anvil. Spectral data were collected using 128 acquisitions with 4 cm^−1^ resolution at 4000–400 cm^−1^. Each sample was measured against an air blank using 128 acquisitions, and three datasets per sample were collected. Spectra were averaged and vectors normalized using the entire frequency range with Bruker OPUS version 7.2 software (Bruker Optik GmbH, Ettlingen, Germany). To separate overlapping absorption bands within the amide I (1700–1600 cm^−1^) and amine II regions (1580–1500 cm^−1^), second derivative spectra were calculated using the Savizky–Golay algorithm with 13 smoothing points, and then vector-normalized using the frequency range 1700–800 cm^−1^.

## Figures and Tables

**Table 1:** Amino acid composition (number of residues/1000 residues) of collagen samples isolated from hoki nasal cartilage.

	Pepsin-Soluble			Alkaline-Soluble		
Amino Acid	Type II	90 kDa	Type IX	Type II	Type IX	Type XI
Asp	50.54	46.44	52.58	51.22	55.19	47.32
Glu	89.27	90.50	86.45	91.68	106.14	98.07
Hyp	105.28	117.73	85.91	96.83	89.38	105.33
Ser	46.40	42.83	46.62	45.45	35.04	36.46
Gly	277.10	286.74	299.31	288.40	278.16	297.04
His	4.16	3.39	7.88	5.11	10.22	6.43
Arg	51.76	43.84	50.22	50.60	43.58	47.20
Thr	26.57	26.34	26.40	25.82	30.16	24.56
Ala	89.46	92.68	74.11	86.12	57.68	65.87
Pro	123.63	122.39	124.60	119.67	93.83	117.94
Tyr	1.63	0.99	1.54	1.98	3.24	1.47
Val	18.47	17.20	24.80	20.74	35.81	22.53
Met	16.48	10.50	13.67	11.77	16.21	12.07
Ile	12.44	11.30	16.02	13.68	25.49	14.10
Leu	41.92	39.57	40.42	41.15	54.45	46.60
Hyl	15.25	17.82	19.48	17.71	28.67	26.28
Phe	14.36	14.76	11.03	14.36	12.01	13.77
Lys	15.28	14.99	17.86	17.46	21.91	16.96
Cys			1.09 ^1^		2.81 ^1^	
Imino (Hyp + Pro)	228.91	240.12	210.51	216.5	183.21	223.27

^1^ Measured value for cysteine is inaccurate, as it is theoretically destroyed during sample preparation.

## Supplementary Materials

The following are available online at https://www.mdpi.com/1660-3397/17/4/223/s1, Figure S1: SDS-PAGE analysis of type II hoki collagen treated with 0.5% pepsin for various times. Lane 1, broad range marker; Lane 2, 0 h; Lane 3, 3 h, Lane 4, 6 h; Lane 5, 24 h. Table S1: Amino acid composition (number of residues/1000 residues) of type II collagen isolated from hoki nasal cartilage and previous studies of type II from other species and tissue. Table S2: Amino acid composition (number of residues/1000 residues) of type IX collagen isolated from hoki nasal cartilage and the previous study of type IX from chicken sternum. Table S3: Amino acid composition (number of residues/1000 residues) of type XI collagen isolated from hoki nasal cartilage and previous studies of type XI from other species and tissue.
