# Supplementary material for: Isolation and Characterisation of Major and Minor Collagens from Hyaline Cartilage of Hoki (Macruronus novaezelandiae)
Source: Mar Drugs. 2019 Apr 12;17(4):223. doi: 10.3390/md17040223 (PMC6521242; doi:10.3390/md17040223)
Supplement: Supplementary file 1 [file marinedrugs-17-00223-s001.pdf]

marine drugs

Supplementary Materials

Isolation and Characterization of Major and Minor Collagens from Hyaline Cartilage of Hoki (*Macruronus Novaezelandiae*)

Mathew H Cumming [1]*, Bronwyn Hall [1] and Kathleen Hofman [1]

Seafood Research Unit, The New Zealand Institute for Plant and Food Research Limited, P.O. Box 5114, Port Nelson, 7043, New Zealand; Bronwyn.hall@plantandfood.co.nz (B.H.); Kathleen.Hofman@plantandfood.co.nz (K.H.)

* Correspondence: Mathew.cumming@plantandfood.co.nz; Tel.: +64-3-989-7645

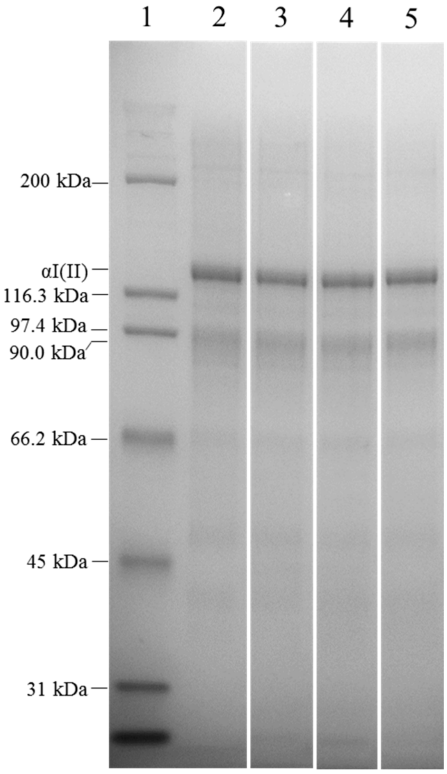

Figure S1. SDS-PAGE analysis of type II hoki collagen treated with 0.5% pepsin for various times. Lane 1, Broad range marker; Lane 2, 0 h; Lane 3, 3 h, Lane 4, 6 h; Lane 5, 24 h.

Table S1. Amino acid composition (number of residues/1000 residues) of type II collagen isolated from hoki nasal cartilage and previous studies of type II from other species and tissue.

	Hoki Pepsin-Soluble Type II	Hoki Alkaline-Soluble Type II	Lamprey Notochord	Skate Fin	Sturgeon Cartilage	Bovine Cartilage	Chicken Sternum
**Asp**	50.5	51.22	47.0	41.0	43.9	48.0	46.0
**Glu**	89.3	91.68	99.0	90.0	88.5	92.0	85.0
**Hyp**	105.3	96.83	74.0	75.0	104.5	97.0	117.0
**Ser**	46.4	45.45	38.0	30.0	37.3	28.0	22.0
**Gly**	277.1	288.4	330.0	352.0	322.9	330.0	310.0
**His**	4.2	5.11	4.0	2.0	8.1	2.0	4.0
**Arg**	51.8	50.6	51.0	51.0	53.0	51.0	52.0
**Thr**	26.6	25.82	27.0	39.0	21.9	23.0	26.0
**Ala**	89.5	86.12	104.0	95.0	89.0	98.0	104.0
**Pro**	123.6	119.67	115.0	113.0	121.6	115.0	115.0
**Tyr**	1.6	1.98	2.0	2.0	2.6	1.0	5.0
**Val**	18.5	20.74	12.0	19.0	15.5	18.0	19.0
**Met**	16.5	11.77	0.0	10.0	3.9	9.0	10.0
**Ile**	12.4	13.68	11.0	8.0	11.5	10.0	11.0
**Leu**	41.9	41.15	29.0	27.0	27.7	26.0	27.0
**Hyl**	15.3	17.71	22.0	18.0	18.7	20.0	-
**Phe**	14.4	14.36	12.0	12.0	14.6	14.0	15.0
**Lys**	15.3	17.46	16.0	12.0	15.3	18.0	14.0
**Cys**	-	-	-	-	-	-	-
**Citation**			[22]	[31]	[43]	[30]	[28]

Table S2. Amino acid composition (number of residues/1000 residues) of type IX collagen isolated from hoki nasal cartilage and the previous study of type IX from chicken sternum.

	Hoki Pepsin-Soluble Type IX	Hoki Alkaline-Soluble Type IX	Chicken Sternum
**Asp**	52.6	55.2	53
**Glu**	86.5	106.1	97
**Hyp**	85.9	89.4	95
**Ser**	46.6	35.0	38
**Gly**	299.3	278.2	323
**His**	7.9	10.2	8
**Arg**	50.2	43.6	46
**Thr**	26.4	30.2	16
**Ala**	74.1	57.7	57
**Pro**	124.6	93.8	90
**Tyr**	1.5	3.2	5
**Val**	24.8	35.8	23
**Met**	13.7	16.2	9
**Ile**	16.0	25.5	27
**Leu**	40.4	54.5	48
**Hyl**	19.5	28.7	34
**Phe**	11.0	12.0	8
**Lys**	17.9	21.9	21
**Cys**	1.1 [1]	2.8 [1]	2
**Citation**			[20]

[1] Measured value for cysteine is inaccurate, as it is theoretically destroyed during sample preparation.

Table S3. Amino acid composition (number of residues/1000 residues) of type XI collagen isolated from hoki nasal cartilage and previous studies of type XI from other species and tissue.

	Hoki Alkaline-Soluble Type XI	Lamprey Notochord α1(XI)	Lamprey Notochord α2(XI)	Human Cartilage α1(XI)	Human Cartilage α2(XI)
**Asp**	47.3	58.0	52.0	46.0	50.0
**Glu**	98.1	105.0	100.0	107.0	98.0
**Hyp**	105.3	61.0	56.0	98.0	93.0
**Ser**	36.5	42.0	43.0	25.0	28.0
**Gly**	297.0	332.0	360.0	334.0	327.0
**His**	6.4	8.0	8.0	6.0	11.0
**Arg**	47.2	42.0	43.0	45.0	48.0
**Thr**	24.6	24.0	28.0	17.0	25.0
**Ala**	65.9	69.0	65.0	54.0	49.0
**Pro**	117.9	106.0	96.0	109.0	119.0
**Tyr**	1.5	2.0	< 2.0	< 2.0	3.0
**Val**	22.5	30.0	25.0	28.0	18.0
**Met**	12.1	-	-	10.0	9.0
**Ile**	14.1	19.0	16.0	15.0	16.0
**Leu**	46.6	46.0	44.0	35.0	39.0
**Hyl**	26.3	31.0	37.0	38.0	40.0
**Phe**	13.8	8.0	9.0	11.0	11.0
**Lys**	17.0	21.0	17.0	19.0	15.0
**Cys**	-	-	-	-	-
**Citation**		[22]	[22]	[33]	[33]
